# Interaction of Sex and Age on the Dissociative Effects of Ketamine Action in Young Healthy Participants

**DOI:** 10.3389/fnins.2019.00616

**Published:** 2019-06-18

**Authors:** B. Derntl, J. Hornung, Z. D. Sen, L. Colic, M. Li, M. Walter

**Affiliations:** ^1^Department of Psychiatry and Psychotherapy, Eberhard Karls University of Tübingen, Tübingen, Germany; ^2^Werner Reichardt Centre for Integrative Neuroscience, University of Tübingen, Tübingen, Germany; ^3^LEAD Research School & Network, University of Tübingen, Tübingen, Germany; ^4^Clinical Affective Neuroimaging Laboratory, Magdeburg, Germany; ^5^Department for Behavioral Neurology, Leibniz Institute for Neurobiology, Magdeburg, Germany; ^6^Max Planck Institute for Biological Cybernetics, Tübingen, Germany; ^7^Center for Behavioral Brain Sciences, Magdeburg, Germany; ^8^Department of Psychiatry and Psychotherapy, Otto von Guericke University Magdeburg, Magdeburg, Germany

**Keywords:** ketamine, dissociation, depersonalization, sex, age, brain maturation

## Abstract

Ketamine is a drug that reduces depressive and elicits schizophrenia-like symptoms in humans. However, it is largely unexplored whether women and men differ with respect to ketamine-action and whether age contributes to drug-effects. In this study we assessed dissociative symptoms via the Clinician Administered Dissociative States Scale (CADSS) in a total of 69 healthy subjects aged between 18 and 30 years (early adulthood) after ketamine or placebo infusion. Dissociative symptoms were generally increased only in the ketamine group post-infusion. Specifically, within the ketamine group, men reported significantly more depersonalization and amnestic symptoms than women. Furthermore, with rising age only men were less affected overall with respect to dissociative symptoms. This suggests a sex-specific protective effect of higher age which may be due to delayed brain maturation in men compared to women. We conclude that it is crucial to include sex and age in studies of drug effects in general and of ketamine-action in specific to tailor more efficient psychiatric treatments.

**Clinical Trial Registration:** EU Clinical Trials Register (EudraCT), trial number: 2010-023414-31.

## Introduction

Ketamine is an N-methyl-D-aspartate (NMDA) receptor antagonist and has been shown to decrease depressive symptoms in humans (Murrough et al., 2013), even for low doses (Xu et al., 2016), leading to rapid acting and long lasting effects. Furthermore, ketamine leads to schizophrenia-like symptoms including positive and negative symptoms and has been used as a psychosis model in both human and animal studies for decades (Krystal et al., 1994; Adler et al., 1998; Newcommer et al., 1999). Additionally, acute ketamine administration induces transient dissociative symptoms, i.e., a kind of experience of detachment from surroundings, body and time (Sleigh et al., 2014). Importantly, ketamine induced dissociative symptoms, especially the degree of depersonalization, can predict the antidepressant response 24 h after ketamine infusion in major depression patients, whereas neither other acute psychotomimetic nor physiological effects can (Luckenbaugh et al., 2014; Niciu et al., 2018).

Despite the long history of ketamine’s use in experimental and clinical medicine, only few studies have addressed the question whether modulatory factors like sex and age may contribute to the effects of ketamine. In animal studies sex-specific effects of repeated ketamine administration have been shown leading to antidepressant effects and enhanced hippocampal synapsin levels in male mice but increased depressive like symptoms and attenuated glutamate and aspartate levels in female mice (Thelen et al., 2016). Other studies reported faster antidepressant effects in female but longer lasting effects in male mice (Franceschelli et al., 2015) and higher sensitivity of female rats to low doses of ketamine (Carrier and Kabbaj, 2013). Furthermore, juvenile males were reported to be less sensitive to antidepressant effects of ketamine in comparison to adult male rats (Parise et al., 2013). Regarding sex-specific effects of ketamine in humans, initial research provides evidence that after drug infusion men show a larger decline of verbal memory than women (Morgan et al., 2006). However, most of the previously published studies did not investigate an effect of sex or the interaction of sex and age on any of the ketamine induced symptoms and physiological alterations, despite its great relevance for uncovering ketamine’s therapeutic potential (Wright and Kabbaj, 2018).

Among many targets, ketamine is primarily an NMDA receptor antagonist and its consequent enhancing effect in the function of another glutamatergic ionotropic receptor, AMPA receptor, is well known (for review see Aleksandrova et al., 2017). The glutamatergic system displays prominent sex differences from the DNA level to physiological behaviors of neurons, potentially contributing to the well-known gap in prevalence rates, symptomatology and treatment success in women and men suffering from mental disorders (for review see Wickens et al., 2018). The little information we have from human studies indicates that women show higher levels of glutamate compared to men, in particular in the striatum and the cerebellum (Zahr et al., 2013) as well as the sensorimotor cortex and the anterior cingulate cortex (Grachev and Apkarian, 2000). Moreover, changes in cerebral glutamate levels across the lifespan have been predominantly reported in adult men, exhibiting a steep decline with age (Sailasuta et al., 2008). Interestingly, serum levels of glutamate increase with older age in adult women, while this is not observed in men (Kouchiwa et al., 2012).

Sex and age effects have been more extensively explored in animal studies, critically pointing to the differential reorganization of the glutamatergic system in the developmental period in females and males especially in the prefrontal cortex (PFC; Spear, 2000). The NR2 subunit of NMDA receptor displays a developmental switch as the NR2B subunits in the PFC play important roles in regulating the maturation of PFC circuits in the transition phase between puberty and early adulthood (Flores-Barrera et al., 2014). Provided that maturation of the PFC is not completed until mid-twenties due to gradual synaptic pruning throughout adolescence and early adulthood (Tsujimoto, 2008; Elston et al., 2010; Kolb et al., 2012), these developmental stages can be called as critical periods (Bale and Epperson, 2017). These critical periods also correspond to the developmental stages, where female and male brains become more and more distinct from each other in many levels (Bale and Epperson, 2017). Men and women differ with respect to brain maturation leading to a 1–2 years earlier peak of gray maturation (Lenroot et al., 2007) as well as reduced cortical gray matter loss during adolescence/early adulthood in women (Sandu et al., 2014). Among other brain regions like the amygdala, hippocampus or hypothalamus, orbital and medial PFC show sexual dimorphisms (Goldstein et al., 2001) and differing maturation processes, e.g., gray matter in frontal cortices becomes thinner earlier in females than males (McEwen and Morrison, 2013). Interestingly, Deakin et al. (2008) showed that dissociative effects of ketamine are associated to activity in ventromedial regions of PFC.

In view of these findings, we expected sex differences with respect to dissociative symptoms after single ketamine infusion in women and men during early adulthood when brain maturation is still ongoing. This study was designed to compare effects of a ketamine infusion in healthy young women and men using the Clinician-Administered Dissociative States Scale (CADSS) assessing dissociative symptoms, i.e., derealization, depersonalization, and amnestic effects. To do so, we matched women and men for age and restricted the age range from 18 to 30 years.

## Materials and Methods

### Participants

The study was part of a randomized, double-blind, placebo-controlled trial (EudraCT number: 2010-023414-31). Participants were recruited by public advertisement. Participants were screened for MR compatibility and completed extensive medical examination to assure healthy physical status. The German version of Mini-International Neuropsychiatric Interview (MINI; Sheehan et al., 1998) was used to exclude DSM–IV psychiatric disorders. Participants were additionally screened for general psychiatric (BPRS; Overall and Gorham, 1960) depressive (HAM-D; Hamilton, 1960) and anxiety related symptoms (HAM-A; Hamilton, 1959). Moreover, participants were free of current substance use or abuse (excluding smoking) and did not take any medication (excluding contraception pills). Seventeen subjects were excluded during screening process. The age range was set to 18–30 years. Thus, 29 healthy female and 40 male participants were recruited and randomly assigned to receive either a racemic ketamine or a placebo (saline) infusion. Study investigators, research coordinators, attending care teams and subjects were blind to treatment allocation. Fourteen women (mean age = 23.43 years; SD = 2.47) and 21 men (mean age = 24.57; SD = 2.51) received ketamine, whereas 15 women (mean age = 24.33 years; SD = 2.66) and 19 men (mean age = 24.00 years; SD = 1.97) received a placebo infusion. The Ethics Committee of the Medical Faculty of the University of Magdeburg approved the experimental protocol of the study and the study was conducted in accordance with the Declaration of Helsinki (World Medical Association, 2002). Participants provided written informed consent prior to participation and received financial compensation for their participation.

### Procedure

First participants completed a baseline assessment of the CADSS (Bremner et al., 1998) which assesses dissociative symptoms divided into depersonalization, derealization, and amnestic symptoms. Afterward, 50 ml of either 0.9% saline (NaCl 0.9%; Berlin-Chemie AG, Berlin, Germany) or 0.5 mg/kg body weight of ketamine +/− racemate (Ketamine-ratiopharm^®^500 mg/10 ml; Ratiopharm GmbH, Ulm, Germany) were infused continuously over 40 min via an infusion pump (Injectomat 2000; Fresenius Kabi GmbH, Langenhagen, Germany). Immediately after the end of infusion, participants completed the CADSS the second time and again 20–40 min after the end of infusion. During infusion, participants were monitored for cardiovascular response every 5 min, and again 20 as well as 60 min after infusion (Liebe et al., 2017). To ensure the safety of participants, they were additionally asked about their general condition after the end of infusion.

### Statistical Analysis

Statistical analyses were conducted via SPSS 23 (IBM). First independent samples *t*- or *U*-tests compared the placebo and ketamine group with respect to demographical variables. Further women and men in the ketamine group were also compared for the same variables.

To assess whether placebo and ketamine group differed for baseline or post-infusion dissociative symptoms, a 2 × 2 independent samples analysis of variance (ANOVA) was conducted including the within-subject-factor Time (baseline, post-infusion) and the between-subject-factor Treatment (placebo, ketamine).

To investigate dissociative symptoms in the ketamine group, a multivariate analysis of covariance (MANCOVA) with sex (women, men) as fixed factor and the three CADSS-subscales (scores after ketamine infusion) as dependent variables was conducted to assess sex differences across all subscales. Test statistics are reported according to Pillai’s Trace. Furthermore, a univariate ANCOVA was computed to assess sex differences for the total score. To adjust for potential differences on the total dose depending on body weight, this variable was added as a covariate to both tests. In each case, the statistical threshold was set to α = 0.05. *P*-values between 0.05 and 0.09 were labeled trend-significant.

### Correlation Analyses

To assess whether age was associated to symptom manifestation for the total score or one of the three subscales within the ketamine group, partial correlations controlling for weight were conducted separately for women and men. To address the question whether correlation coefficients were different for women and men, Fisher’s *z* tests were computed. Differences in correlation scores were corrected for multiple comparisons, with an effective threshold of *p* < 0.0125.

## Results

### Demographics

Independent samples *t*-tests confirmed that the placebo and ketamine group did not differ significantly in demographic variables or psychiatric symptoms. Also, women and men in both groups did not differ in all parameters except weight (see Table 1).

**Table 1:** Demographic details of the ketamine and placebo group for female and male participants.

	Ketamine (*n* = 35)		Sex-differences	Placebo (*n* = 34)		Sex-differences	Treatment group -differences
Sex	14 F/21 M			15 F/19 M			χ^2^(1) = 0.12, *p* = 0.73
Age	24.11 (2.53)			24.15 (2.27)			*U* = 587, *p* = 0.92
	M	24.57 (2.52)	*t*(33) = −1.32, *p* = 0.20	M	24.0 (1.97)	*U* = 130.5, *p* = 0.68	
	F	23.43 (2.47)		F	24.33 (2.66)		
BMI	23.82 (3.07)			23.57 (3.01)			*U* = 539, *p* = 0.72
	M	24.15 (2.98)	*t*(33) = −0.77, *p* = 0.45	M	23.80 (2.50)	*U* = 115, *p* = 0.35	
	F	23.33 (3.26)		F	23.28 (3.62)		
Weight (kg)	74.65 (13.88)			74.37 (14.50)			*t*(67) = 0.08, *p* = 0.94
	M	81.71 (12.58)	*t*(33) = −4.68, *p* < 0.001	M	81.03 (12.99)	*U* = 41, *p* < 0.001	
	F	64.07 (7.72)		F	65.93 (11.91)		
HAM-A	0.63 (1.11)			0.26 (0.79)			*U* = 478, *p* = 0.063
	M	0.71 (1.31)	*U* = 147, *p* > 0.9	M		*U* = 129, *p* = 0.66	
	F	0.50 (0.76)		F	0.40 (1.06)		
HAM-D	0.51 (1.10)			0.24 (0.55)			*U* = 539, *p* = 0.35
	M	0.62 (1.28)	*U* = 136, *p* = 0.73	M	0.16 (0.50)	*U* = 120.5, *p* = 0.45	
	F	0.36 (0.75)		F	0.33 (62)		
CADSS total score baseline	–			–			
CADSS total score end of infusion	9.46 (8.76)			0			*F*(1,67) = 39.65, *p* < 0.001, η^2^ = 0.37
	M	10.57 (8.23)	*F*(1,32) = 2.99, *p* = 0.09, η^2^ = 0.09				
	F	7.79 (9.56)					
CADSS total score 20–40 min end of infusion	–			–			

*Both groups did not differ in age and psychiatric symptoms. Only in the ketamine group general CADSS scores showed an increase post-infusion, that differed between men and women Variables are presented as mean (SD). Hamilton Anxiety Rating Scale (HAM-A); Hamilton Depression Scale (HAM-D), Clinician Administered Dissociative Symptoms Scale (CADSS), F (females), M (males).*

### General Effects of Ketamine Infusion

First, a 2 × 2 ANOVA detected main effects of Treatment, *F*(1,67) = 39.65, *p* < 0.001, η^2^ = 0.37, and Time, *F*(1,67) = 39.65, *p* < 0.001, η^2^ = 0.37, and a significant interaction of Treatment × Time, *F*(1,67) = 39.65, *p* < 0.001, η^2^ = 0.37, indicating enhanced dissociative symptoms only in the ketamine group immediately post-infusion.

### Sex-Specific Effects in the Ketamine Group Depending on CADSS Subscale

**FIGURE 1:**
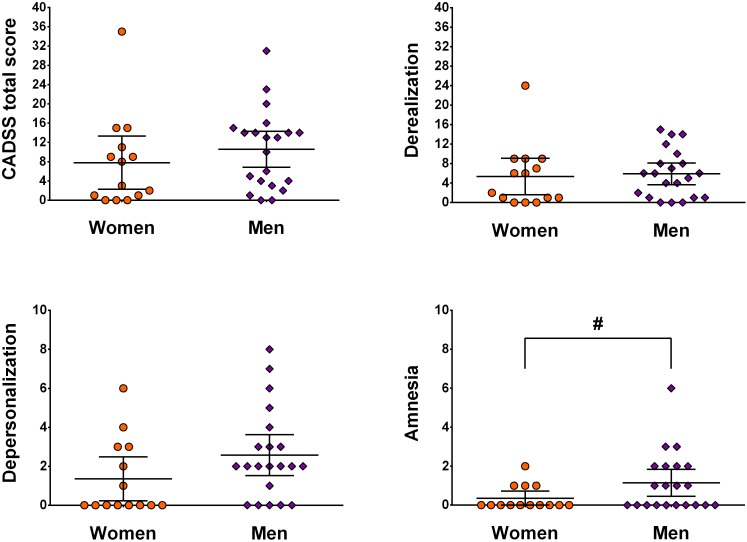
Illustration of sex- and subscale-specific effects of ketamine. Data is presented as mean ± 95 CI and is not corrected for weight. ^#^ indicates *p* < 0.05.

Second, sex- and subscale-specific effects of ketamine were investigated. The MANCOVA including weight as covariate showed a significant effect of sex [*F*(3,30) = 3.60, *p* = 0.025, η^2^ = 0.27]. Looking at subscales individually, depersonalization [*F*(1,32) = 7.38, *p* = 0.011, η^2^ = 0.19] and amnesia [*F*(1,32) = 5.09, *p* = 0.031, η^2^ = 0.14] showed significantly higher scores in men, which was not the case for derealization [*F*(1,32) = 0.93, *p* = 0.34, η^2^ = 0.03]. Univariate ANCOVA showed a marginal effect on the total score [*F*(1,32) = 2.99, *p* = 0.09, η^2^ = 0.09] (see Figure 1).

### Sex- and Subscale Specific Effects in Connection With Participant Age

**FIGURE 2:**
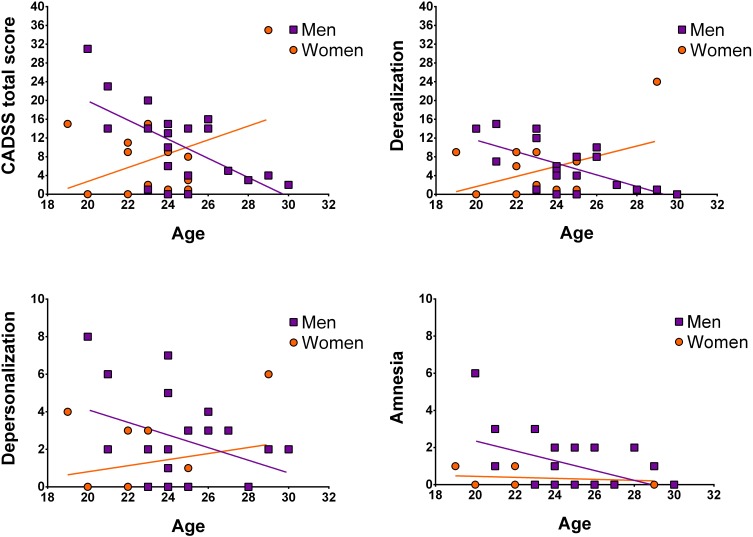
Illustration of correlations between age and CADSS scores separately for women and men. Data points are not corrected for weight.

Next, we assessed whether participant age was associated with symptom manifestation in CADSS total and sub-scores, separately for men and women. In men, significant negative correlations between age and CADSS scores were observed (for depersonalization on a trend-level), which was not the case for women (see Figure 2 and Table 2).

**Table 2:** Correlation analyses testing sex-specific associations between Age and CADSS scores immediately after infusion.

	Men (*n* = 21)	Women (*n* = 14)	Fisher’s *z* (*p*-value) sex-difference
Age × CADSS total score	−0.64 (0.002)	0.36 (0.22)	−**2.99 (0.003)^∗^**
Age × CADSS derealization	−0.64 (0.002)	0.40 (0.18)	−**3.08 (0.002)^∗^**
Age × CADSS depersonalization	−0.39 (0.085)	0.19 (0.53)	−1.59 (0.11)
Age × CADSS amnesia	−0.45 (0.048)	−0.11 (0.73)	−0.98 (0.33)

*Women and men differed with respect to the CADSS total score and the subscale derealization. Clinician Administered Dissociative Symptoms Scale (CADSS). Data is presented as r coefficient (p-value). Bold values and ^∗^indicate p < 0.05.*

To further validate whether men and women differed for any of the above correlations, Fisher’s *Z*-tests were conducted. For derealization, *z* = −3.08, *p* = 0.002, and the CADSS total score, *z* = −2.99, *p* = 0.003, significant sex-differences were detected indicating that in men symptom manifestation decreased more strongly with age than in women. Correlation coefficients of men and women did not differ significantly for depersonalization z = −1.59, *p* = 0.11 and amnesia, *z* = −0.98, *p* = 0.33 (see Table 2).

## Discussion

The present study investigated whether dissociative symptoms as induced by the anti-depressive drug ketamine differ as a function of sex and age. In general, a sub-anesthetic dose of ketamine led to profound dissociate symptoms affecting women and men, though men showed significantly stronger symptom manifestation regarding depersonalization and amnesia than women. Furthermore, taking into account our participants age, in men dissociative symptoms in total and derealization in specific decreased with rising age while this association was not observed in women.

Surprisingly, the effects of sex and age on ketamine’s actions have not been broadly examined in humans, although prevalence rates and symptomatology of mental disorders associated with the glutamate system and ketamine-action, e.g., depression, significantly differ between women and men (Whiteford et al., 2013; Strong and Kabbaj, 2018; Wickens et al., 2018; Wright and Kabbaj, 2018). Although animal studies point to a variation in sensitivity to antidepressant and addictive effects of ketamine depending on age and sex (Carrier and Kabbaj, 2013; Parise et al., 2013; Franceschelli et al., 2015; Zanos et al., 2016; Schoepfer et al., 2017; Strong et al., 2017), human studies rarely report sex or age effects (Cho et al., 2005; Niciu et al., 2014). In humans, the reported differences between women and men focused on metabolites and hepatic clearance of ketamine (Saland et al., 2017), biomarkers (Colic et al., 2019) or side effects (Liebe et al., 2017). Sigtermans et al. (2009) reported that S-Ketamine is metabolized faster in female subjects and the effect of ketamine is greater on cardiac output and heat pain. Another study which used racemic ketamine, as also used in the current study indicated a sex-specific metabolism of ketamine in depressed and bipolar patients (Zarate et al., 2012). Additionally, a previous meta-analysis reported a significant association between effect sizes of ketamine response at later time points, i.e., 7 days post-infusion, and percentage males, but the number of included studies that contributed data was quite low (Coyle and Laws, 2015). Reviewing the relevant literature, Wright and Kabbaj (2018) stressed that most of the clinical studies lack the information about sensitivity to the effects of ketamine because generally one particular dose of ketamine is administered instead of an application of a dose-response regime like in animal studies. Indeed, Morgan et al. (2006) showed that men are more sensitive to verbal and subjective memory disturbances induced by intravenous ketamine infusions, of which doses ranged between 0.5 and 1.3 mg/kg. Likewise, in the current study male participants reported higher subjective memory disturbances measured by CADSS supporting earlier findings by Morgan et al. The single dose regime that was applied in the current study might have hindered the clear sex effect insensitivity to the amnesic effect of ketamine. More studies using a wider range of doses would be beneficial to understand both the role of sex and age in effects of ketamine.

Concerning age effects, dissociative effects of ketamine were negatively associated with age only in male participants. Early adulthood is a critical development stage that engenders vulnerability for a variety of mental disorders for women and men (Paksarian et al., 2016). Plenty of previously reported findings indicate that the effects of ketamine show variation across participants according to the basal status of associated circuits (Lahti et al., 2001; Corlett et al., 2006; Morgan et al., 2008). Regarding clinical populations, reports on geriatric patients are scarce but seem to be similar to generally observed effects (although see Szymkowicz et al., 2014), but the samples were very small or case-control studies (Iglewicz et al., 2015; George et al., 2017; Medeiros da Frota Ribeiro and Riva-Posse, 2017). Regarding depressed adolescents and young adults, studies investigating antidepressant effects of ketamine are virtually non-existent.

This study is limited in that we specifically focused on young adults, thus included only data from participants younger than 30 years. However, to fully test our assumption of an age-specific decline of dissociative symptoms in men, future studies should include a broader age range, informing about age- and sex-specific effects across different developmental stages (e.g., from puberty to menopause and further) as changes across the lifespan have been reported for cerebral glutamate levels in men (Sailasuta et al., 2008) and serum levels in women (Kouchiwa et al., 2012). Moreover, a modulatory role of estradiol has been shown in animal studies addressing glutamate transmission (Smejkalova and Woolley, 2010). Regarding ketamine, sex differences in ketamine pharmacokinetics in rats have been reported, however, the impact of circulating hormone levels was negligible (Saland and Kabbaj, 2018). Another limitation to be addressed is the lack of measurements of ketamine and its active metabolites in the blood. Evidence indicates that the metabolism of both racemic and S-ketamine differ between men and women (Sigtermans et al., 2009; Zarate et al., 2012). A common limitation of placebo-controlled ketamine studies is the reliability of blinding. Ketamine induces symptoms that are evident mostly to the participants and also to the involved scientists. For this reason, studies are conducted with active placebos like midazolam (Wilkinson et al., 2019), which result in their own caveats.

In summary, male participants in our study reported stronger depersonalization and amnestic symptoms following ketamine infusion. Interestingly, this effect was potentiated by age, i.e., the younger the age the stronger the symptoms. Thus, our findings suggest a sex-specific protective effect of age, which may be due to progressed brain maturation in women compared to men. We conclude that it is crucial to include sex and age in studies of drug effects in general and of ketamine-action in specific to tailor more efficient psychiatric treatment strategies.

## Data Availability

The datasets generated for this study are available on request to the corresponding author.

## Ethics Statement

Participants provided written informed consent prior to participation, received financial compensation for their participation, and the Ethics Committee of the Medical Faculty of the University of Magdeburg approved the experimental protocol of the study.

## Author Contributions

BD, JH, and ZS analyzed the parts of the data and wrote the first draft. LC and ML collected the data, contributed to the data analyses, and corrected the manuscript. MW designed the study, supervised the data collection and analyses, and corrected the manuscript.

## Conflict of Interest Statement

MW received research support from HEEL and Janssen Pharmaceutical Research for a clinical trial on ketamine in patients with major depression which were not investigated in this manuscript. Other authors declare no conflict of interest. The remaining authors declare that the research was conducted in the absence of any commercial or financial relationships that could be construed as a potential conflict of interest.
